# Foregut Duplication Cysts in a Patient With Congenital Diaphragmatic Hernia: A Case Report

**DOI:** 10.7759/cureus.86349

**Published:** 2025-06-19

**Authors:** Aleksandra I Sadecka, Zaneta Slowik-Moczydlowska

**Affiliations:** 1 Departament of Pediatric Surgery, Medical University of Warsaw, Warsaw, POL

**Keywords:** congenital diaphragmatic hernia (cdh), diaphragmatic reconstruction, esophageal duplication, foregut duplication cysts, preterm neonate

## Abstract

Esophageal duplication cysts are a rare form of foregut developmental abnormality with heterogeneous manifestations due to their variable location and size. Congenital diaphragmatic hernia is a congenital defect resulting from incomplete diaphragm formation, with a wide spectrum of severity. It is typically diagnosed antenatally and managed surgically in the neonatal period. We present a case report of a newborn with a rare co-occurrence of congenital diaphragmatic hernia and multiple esophageal duplication cysts.

## Introduction

Duplication cysts of the gastrointestinal tract are rare developmental abnormalities of the foregut, characterized by a heterogeneous clinical presentation due to their variable location and size. Esophageal duplication cysts represent one of the less common forms of this malformation, and due to their frequently asymptomatic nature, they are often diagnosed incidentally [1,2]. Gastrointestinal duplications occur with a frequency of one in 4,500 live births, and approximately 20% of them are esophageal duplications [3]. Congenital diaphragmatic hernia (CDH) is a congenital defect resulting from an incomplete or absent diaphragm. It has a wide spectrum of severity and is usually diagnosed antenatally and managed surgically in the neonatal period. The incidence of CDH ranges from one in 2000 to one in 10,000 live births, and in approximately 40% of cases is associated with other congenital anomalies, such as gastrointestinal duplication, omphalocele, bronchopulmonary abnormalities such as pulmonary sequestration, or esophageal atresia [4-10]. The coexistence of multiple esophageal duplication cysts with congenital diaphragmatic hernia is an exceptional and scarcely reported finding.

## Case presentation

**Figure 1:**
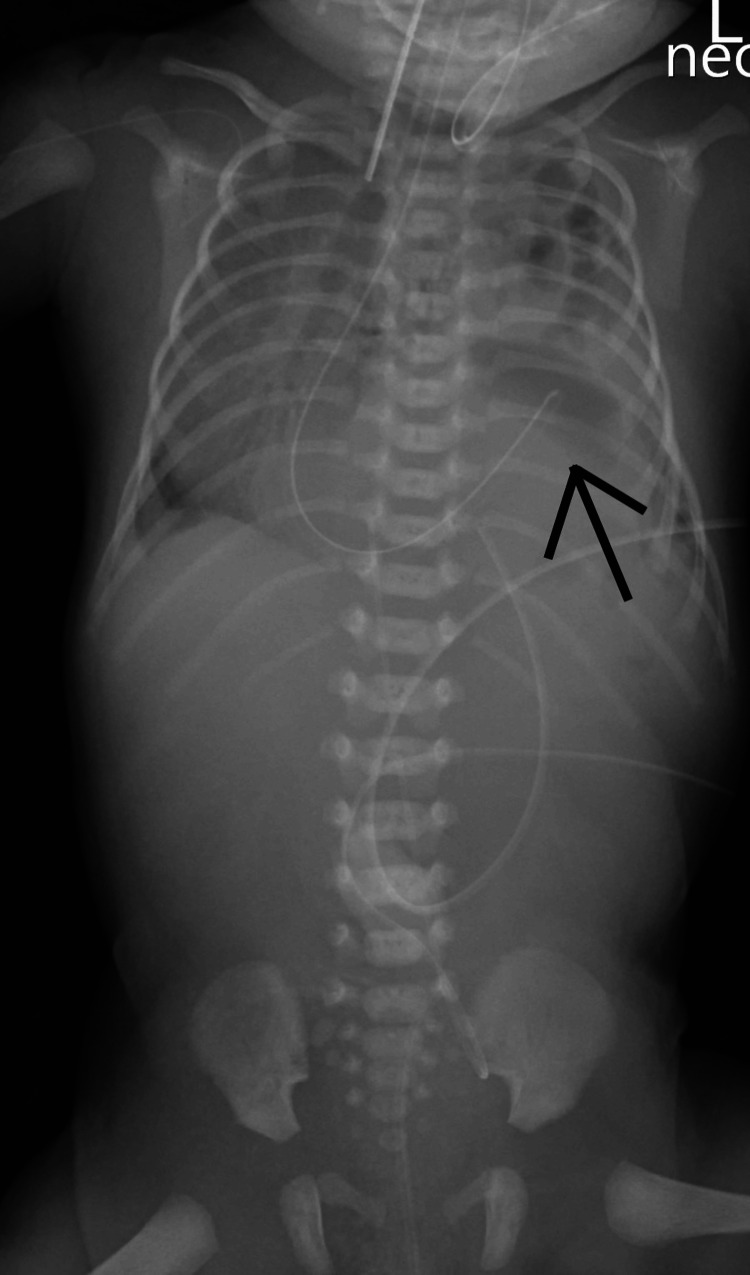
The X-ray before diaphragmatic reconstruction The arrow points at the herniated bowel loops and stomach within the left hemithorax. Additionally, the chest X-ray shows a mediastinal shift.

A preterm female neonate was born at 31 weeks of gestation with a birth weight of 1500 gm and a prenatal diagnosis of CDH. The patient required high-frequency oscillatory ventilation and hemodynamic support with catecholamines. Radiological assessment, including chest and abdominal X-ray, confirmed a left-sided CDH without other abnormalities (Figure 1). Echocardiography was performed and revealed no abnormalities.

**Figure 2:**
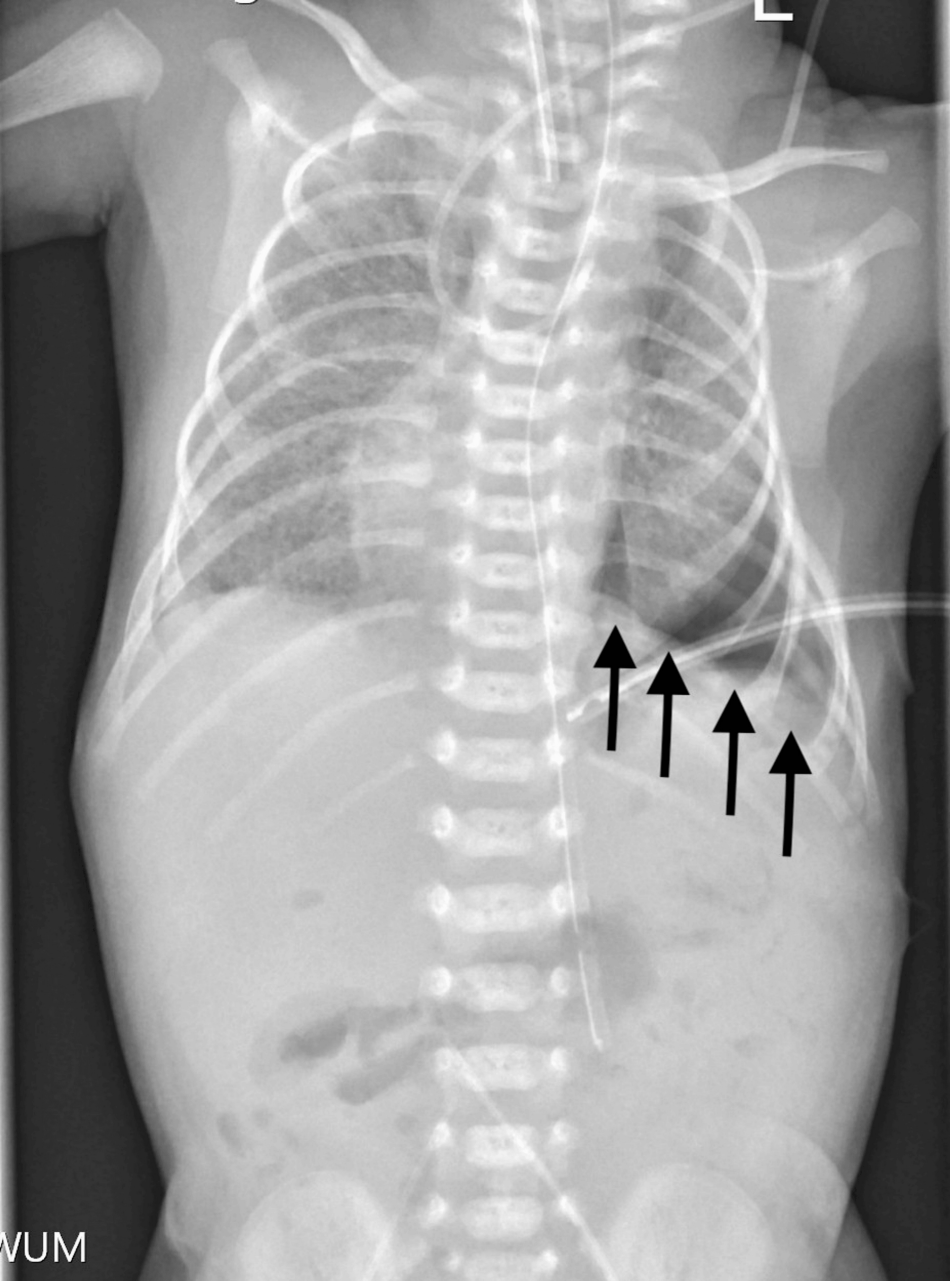
The X-ray after diaphragmatic reconstruction The arrows point to the reconstructed diaphragm.

After initial cardiopulmonary stabilization and correction of fluid and electrolyte imbalances, the patient underwent surgery on the fourth day of life. A laparotomy was performed via a left subcostal incision. During diaphragmatic reconstruction using a GoreTex patch (W.L. Gore & Associates Inc., Newark, DE, USA), two esophageal duplication cysts and one gastric duplication cyst, each measuring 3 cm in diameter, were identified (Figure 2).

**Figure 3:**
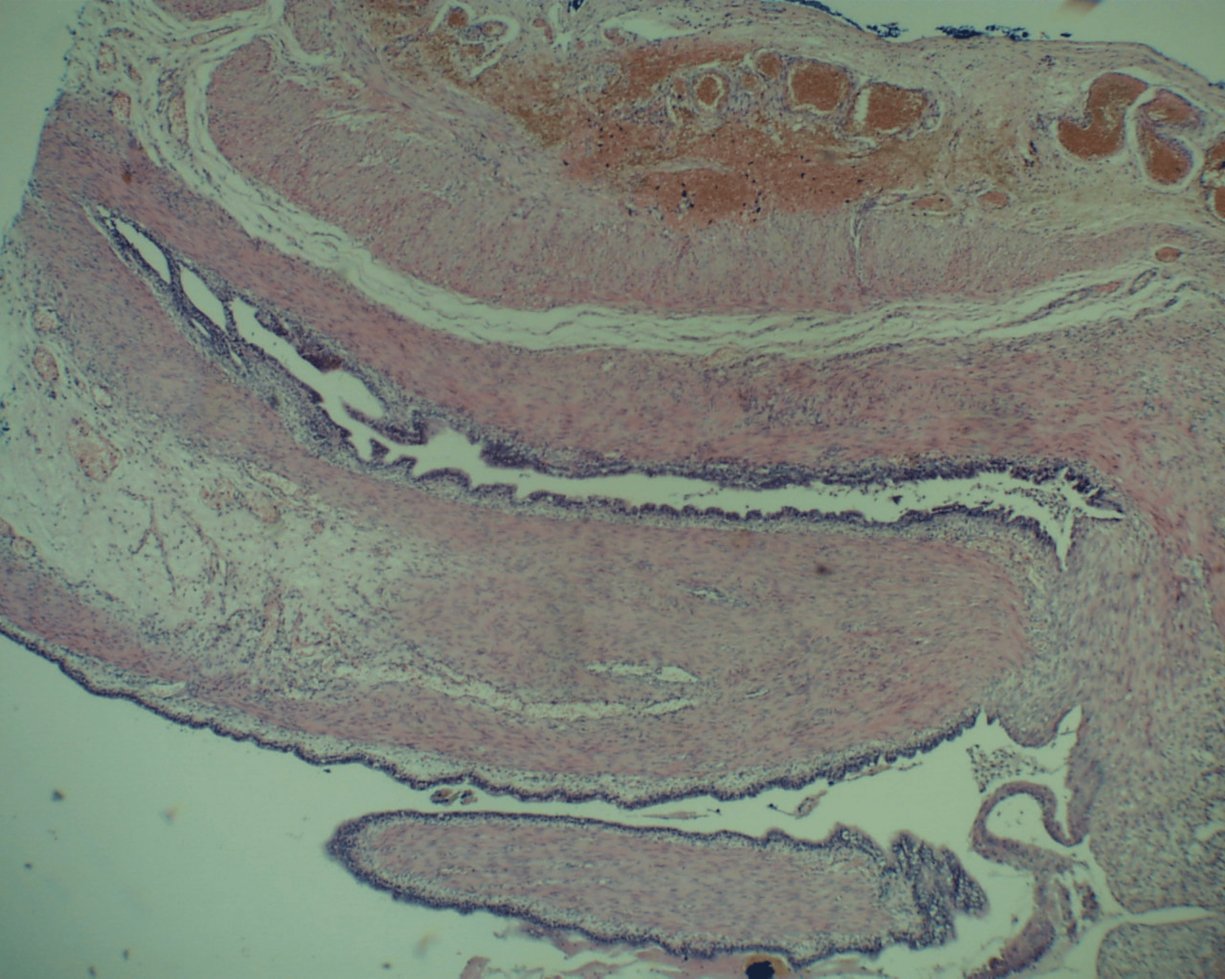
Histopathological examination of the first esophageal duplication cyst

**Figure 4:**
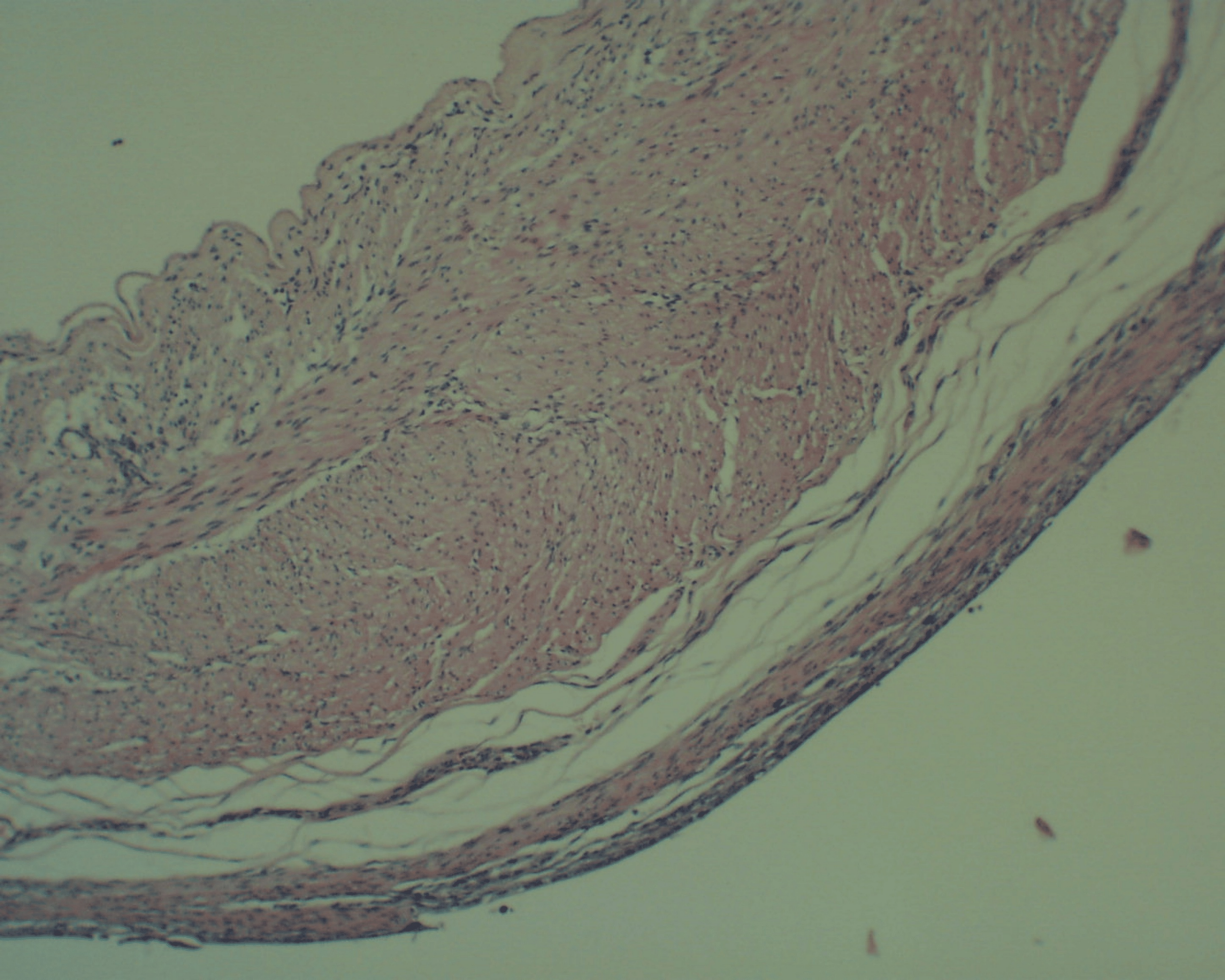
Histopathological examination of the second esophageal duplication cyst

**Figure 5:**
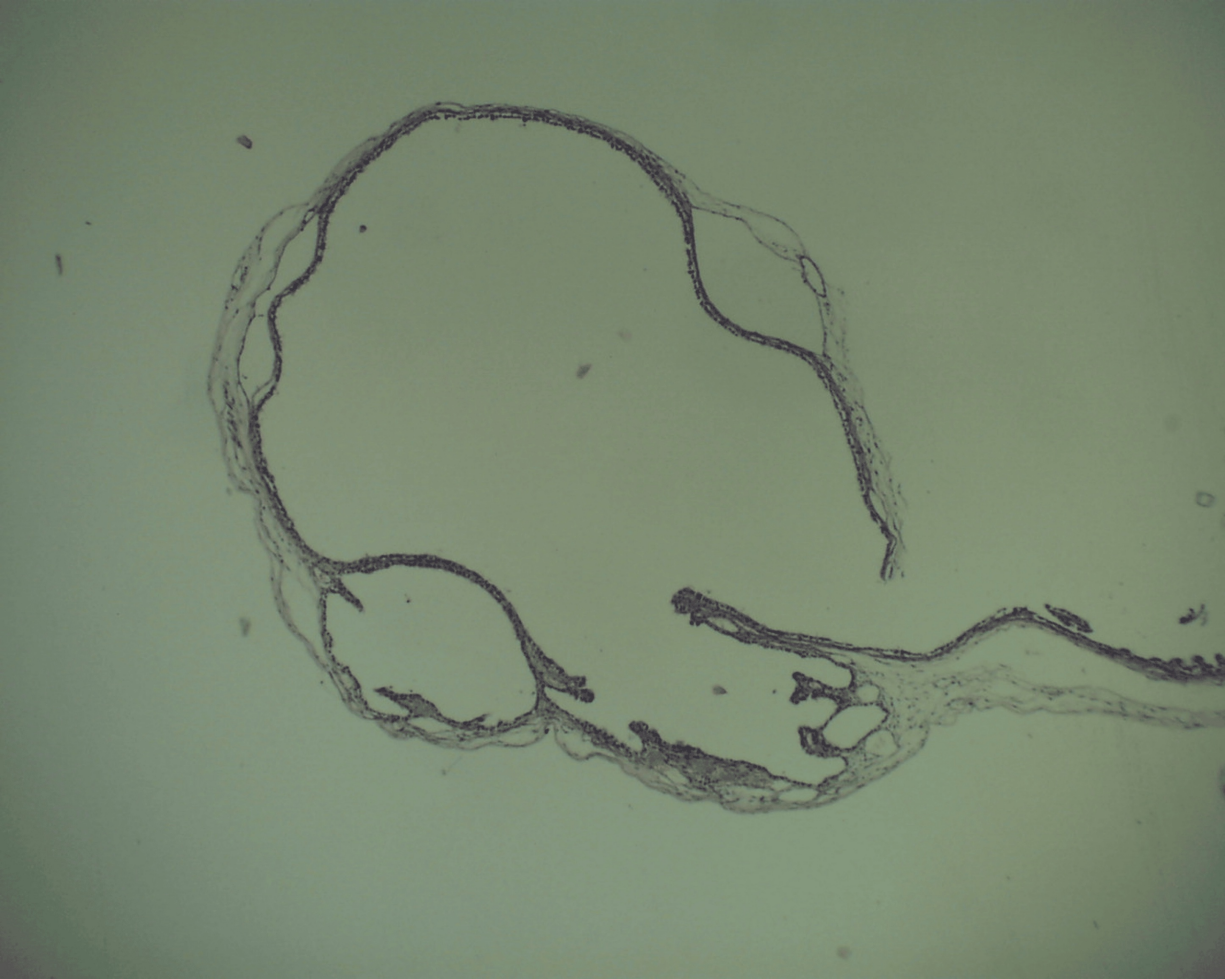
Histopathological examination of the gastric duplication cyst

The cysts had no communication with the esophageal or gastric lumen and were completely enucleated. Histopathological examination confirmed the clinical diagnosis of foregut duplication cysts (Figures 3-5). The postoperative course was complicated by lung hypoplasia and severe right ventricular failure due to pulmonary hypertension, and the patient died on the seventh day of life. 

## Discussion

Congenital diaphragmatic hernia is usually diagnosed antenatally and confirmed postnatally via radiological imaging, typically with an X-ray. Esophageal duplication cysts can be detected prenatally, but the diagnosis is most often made after birth (commonly via CT imaging) or incidentally during a procedure performed for another indication. In some cases, the cyst may be missed during the initial surgery. Gutiérrez reported a case of a neonate with antenatally suspected CDH who underwent a postnatal CT scan. The scan excluded diaphragmatic discontinuity and revealed one mass in the right paravertebral area and another in the posterior mediastinum. The patient underwent staged surgical management: initially, a right thoracotomy was performed to excise the larger mass, followed by a second surgery for the remaining cyst three months later. Gutiérrez concluded that esophageal duplication cysts should be considered in the differential diagnosis of patients with suspected CDH [11]. Seçil et al. presented a case of a four-month-old patient with coexisting CDH, esophageal duplication, and polysplenia. This patient’s CT scan showed a left CDH and a mediastinal mass in the right pleural cavity. The esophageal duplication cyst was solitary and managed surgically during diaphragmatic hernia repair. However, the report lacks details on the cyst's communication with the esophagus and the patient’s postoperative course [12]. In our patient, a CT scan was not performed prior to surgical treatment, as X-ray provides sufficient diagnostic information in cases of CDH without suspicion of additional congenital anomalies on prenatal imaging and echocardiography. Moreover, performing a CT scan in this case would have required general anesthesia and transport to the radiology department, which was not feasible due to the patient’s cardiorespiratory instability.

Coexisting malformations can be missed during CDH repair, and this can cause different symptoms, such as trouble with feeding, and potentially require additional surgery. Danzer et al. reported a case of a patient with a prenatally diagnosed CDH. The hernia was repaired on the sixth day of life. Postoperatively, the patient developed symptoms resembling gastroesophageal reflux (GER). Even though GER is relatively common in children following diaphragmatic repair, further radiological investigations were conducted. An ultrasound scan suggested the hernia’s recurrence, which was excluded by a gastrointestinal contrast study. A CT scan revealed a large cystic mass located behind the esophagus and stomach. The patient underwent another surgical procedure for the excision of a non-communicating esophageal duplication cyst [13].

Unlike congenital diaphragmatic hernia, esophageal duplication is often an asymptomatic anomaly, although it may present with significant clinical complications. Fuchs et al. reported a case of a full-term newborn with significant feeding difficulties manifested by vomiting, which led to aspiration pneumonia. The patient underwent a fundoplication without improvement. A more detailed diagnostic evaluation, including X-ray, bronchoscopy, bronchography, and gastroscopy, failed to identify the underlying cause of the symptoms. The patient was eventually qualified for surgical intervention, which revealed an esophageal duplication associated with esophageal stenosis, congenital diaphragmatic hernia, and pericardial aplasia [14].

Isolated esophageal duplication requires surgical intervention, most commonly performed via thoracotomy or thoracoscopy. However, if the anomaly is discovered incidentally during another procedure, such as CDH repair surgery, it may be managed via another surgical approach, for example, left subcostal laparotomy. Kumar et al. reported a case describing the excision of multiple esophageal duplications via left thoracotomy [15]. Cocker et al. described the resection of multiple esophageal duplication cysts in a 20-month-old patient using a combined thoracoscopic and laparoscopic approach [16]. 

## Conclusions

Because CDH coexists with other congenital anomalies in approximately 40% of cases, surgical repair should include thorough inspection of both the thoracic and abdominal cavities to detect and repair any additional congenital anomalies. Delayed diagnosis and the potential need for subsequent surgical intervention may be significantly more challenging and associated with an increased risk of complications compared to correction during the initial operation.
